# Coronary artery bypass grafting following Ross procedure in a patient with familial hypercholesterolemia

**DOI:** 10.21542/gcsp.2025.49

**Published:** 2025-10-31

**Authors:** Jessica B. Briscoe, Shivani Shirodkar, AlleaBelle Bradshaw, Michelle Carvajal, Jennifer S. Lawton, Hamza Aziz

**Affiliations:** Division of Cardiac Surgery, Department of Surgery, Johns Hopkins University School of Medicine, Baltimore, MD, USA

## Abstract

Patients with familial hypercholesterolemia and concurrent aortic valve disease are at exceptionally high risk of requiring repeat interventions due to coronary disease, in addition to management of valvular pathology. For effective management, it is important for these patients to have a multidisciplinary approach to their care with surgical and interventional cardiology input. In this report, we present a case of a 33-year-old female patient with a history of familial hypercholesterolemia and congenital aortic stenosis status post-Ross procedure who presents with unstable angina. We discuss the challenges and factors to consider in the management of this unique patient population.

## Introduction

Redo sternotomy for cardiac procedures in patients following the Ross procedure is challenging and is associated with risk of injury to the neo aorta, the pulmonic homograft, and the coronary buttons. The decision to perform a Ross procedure in a young patient with familial hypercholesterolemia should be weighed with the potential need for subsequent redo sternotomy for coronary artery bypass grafting (CABG). Patients with familial hypercholesterolemia (FH) have accelerated rates of coronary atherosclerosis. Approximately 30% have coronary artery disease (CAD), and they have an 87% higher risk of experiencing cardiovascular events and death^1,2^.

The Ross procedure is peformed in a few centers and infrequently, given its technical complexity and need for highly experienced surgeons. This has made its utility controversial, as patient selection is critical to mitigate poor outcomes. In addition, Ross historically has been performed on young patients, making a concurrent CABG in this patient population unusual. We describe a young patient with history of familial hypercholesterolemia who underwent CABG 13 years after a Ross procedure which, to the best of our knowledge, has not been previously reported.

## Case presentation

A 33 year old female with FH presented with unstable angina. Her medical history was notable for Ross procedure (#29 CryoLife homograft conduit in pulmonic position) at age 20 for congenital aortic stenosis, CAD and non-ST segment elevation myocardial infarction (NSTEMI), treated 11 months earlier with percutaneous coronary intervention (PCI) involving stenting of the left anterior descending (LAD) and left circumflex (LCx) arteries. Of note, her mother and father had premature CAD, and her brother required a CABG at 24 years of age. The patient’s BMI was 34 kg/m^2^. A stress test was negative for inducible ischemia. The patient’s lipid-lowering regimen consisted of atorvastatin and ezetimibe, which led to her lipid profile markedly improving over the previous year, with her low density lipoprotein cholesterol (LDL-C) reduced to 64 mg/dL (from 443 md/dL).

Preoperative cardiac catheterization demonstrated 70% proximal stenosis at the LAD stent, 80–90% ostial stenosis at the first diagonal branch (D1) and proximal first obtuse marginal branch (OM1) of the circumflex artery, and 70% ostial stenosis of the right coronary artery (RCA). Transthoracic echocardiogram (TTE) showed a left ventricular ejection fraction (LVEF) of 60–65%, Mitral E (velocity of blood from the left atrium (LA) to the left ventricle (LV)) to LV E’ Septal Ratio of 17.63, consistent with elevated left ventricular end diastolic pressure (LVEDP) of 20–25 mmHg. There was no hemodynamically significant aortic stenosis (mean gradient 8.5 mmHg), however, there was mild to moderate central neo-aortic regurgitation and a structurally normal pulmonic valve homograft. The left atrium (LA) was mildly dilated, the right ventricular (RV) systolic pressure was 25 mmHg with normal right atrial (RA) pressure. Given the patient’s history of FH, persistent angina despite previous PCI, and extensive multivessel coronary disease, surgical revascularization with CABG was indicated.

**Figure 1.**
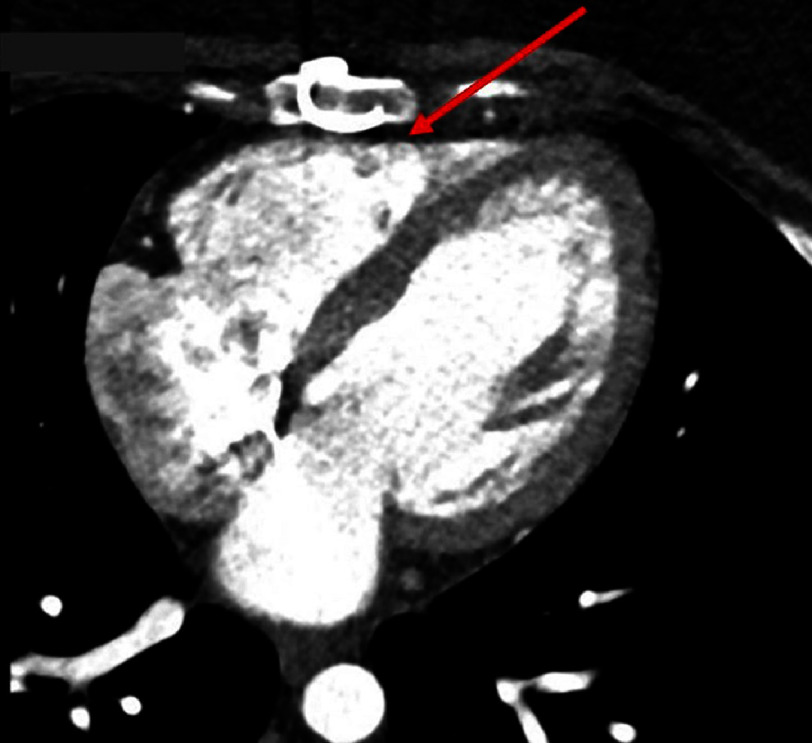
Axial image of computed tomography (CT) scan of chest with contrast demonstrates right ventricular wall near the posterior table of the sternum.

The patient was taken for a redo sternotomy and 3-vessel CABG with the left internal mammary artery (LIMA) to the LAD, right internal mammary artery (RIMA) as a T graft off of the LIMA to the OM, and saphenous venous graft (SVG) from the aorta to the posterior descending artery (PDA). Recognizing the heightened risk of a redo sternotomy, the surgical team instituted preemptive femoral vessel exposure to permit rapid initiation of cardiopulmonary bypass if significant bleeding or cardiac injury were encountered. During the sternotomy, dark venous blood was noticed, suggestive of RV injury, which prompted packing of the sternum. The femoral cannulas were placed and cardiopulmonary bypass was initiated. Upon continuation of the sternotomy, injury of the RV outflow tract was noted, which was the prior homograft densely adhered to the sternum (Figure 1).

Management of intraoperative bleeding included packing the mediastinum, establishing cardiopulmonary bypass, and then executing primary suture repair of the right ventricular outflow tract. Despite control of the direct injury, the patient exhibited profound coagulopathy. The patient was managed with 8 units of packed red blood cells, 12 units of fresh frozen plasma, 5 units of platelets, 4 units of cryoprecipitate, and return of 5.5 liters of autologous blood via cell saver, in addition to 10 liters of crystalloid administered and a urine output of 3.5 liters.

Given the constraints posed by dense adhesions and increased risk of RV injury, together with the PDA’s small caliber (1.75–2 mm), vein grafting was considered most suitable for achieving reliable revascularization. The CABG portion of the procedure was uneventful. Due to coagulopathy, her chest was left open and closed on postoperative day (POD) 1 with bilateral pectoralis muscle flaps performed by the plastic surgery team to augment soft tissue over the mediastinum, provide vascularized coverage, and reduce the risk of infection in the context of delayed sternal closure.

The post-operative course was complicated by dysphonia, and she was found to have a vocal cord polyp. She was discharged home on POD 11. PCSK9 inhibitor and bempedoic acid/ ezetimibe combination therapy was added to her anti-dyslipidemic regimen in the outpatient setting. Unfortunately, the patient was readmitted on POD 18 for chest pain, abdominal pain, and diarrhea. During her admission, *C. difficile* was negative, pantoprazole was stopped, and diarrhea improved. On repeat echocardiography, LVEF was estimated at 60–65%, with moderate aortic regurgitation, a mildly increased gradient across the aortic valve, mild-moderate tricuspid regurgitation, a mildly increased gradient across the pulmonic valve replacement (peak 32 mmHg), and a left pleural effusion that did not warrant treatment. The patient reported palpitations and was found to have paroxysmal episodes of atrial fibrillation. She was initiated on rivaroxaban for anticoagulation and discharged with a cardiac event monitor on POD 20.

**Figure 2.**
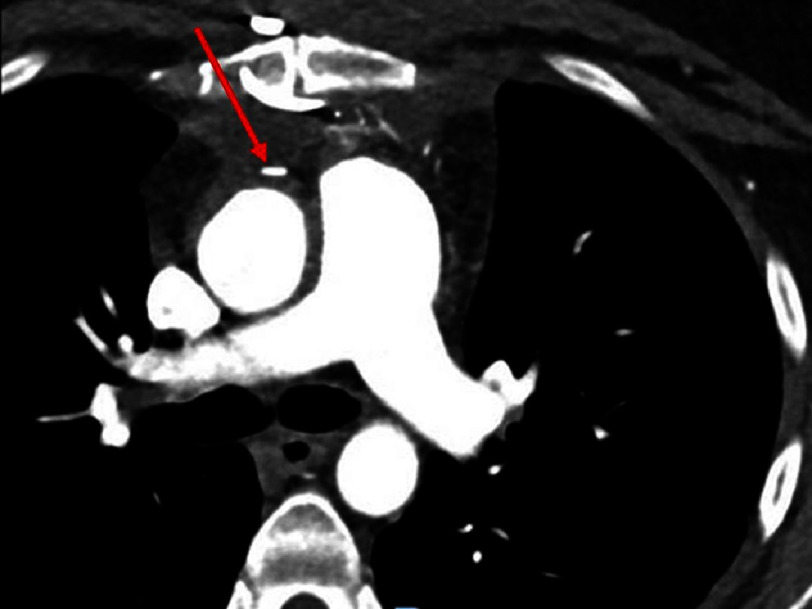
Axial image of computed tomography scan of chest (coronary CT) at the level of the pulmonary bifurcation demonstrates radiopaque marker anterior to the ascending that marks the proximal anastomosis. There is no contrast seen in the saphenous vein graft (SVG) originating from the aorta at this location that was sewn to the posterior descending artery (PDA) during coronary artery bypass grafting (CABG).

At the patient’s routine cardiology visit on POD 48, the patient reported intermittent angina, not always related to physical activity, and intermittent palpitations. The coronary CT scan images demonstrated occlusion of the SVG-PDA graft. (Figure 2).

Ongoing symptoms and angiographic evidence of further disease resulted in multiple subsequent coronary interventions: percutaneous coronary intervention to the right coronary artery 17 weeks after CABG, stenting of the distal right internal mammary artery to obtuse marginal anastomosis in week 18 and repeat PCI to the LIMA–RIMA anastomosis approximately 40 weeks after the surgery. Her lipid profile improved due to adherence to her medications, with her LDL-C levels 53 mg/dL and her cholesterol being 99mg/dL. Across these interventions, the patient experienced partial, sustained improvement in symptoms with increased physical activity.

## Discussion

FH is an autosomal dominant mutation characterized by increased total cholesterol and low-density lipoprotein (LDL) over a patient’s lifetime. Traditionally, FH is associated with diffuse vascular atherosclerosis; however, recent evidence also shows associated aortic root and aortic valve stenosis^3,4^. Typical complications of the Ross procedure involve neo-aortic or pulmonary conduit stenosis or dilation. However, despite coronary reimplantation, coronary compromise is not a frequent indication for reintervention^5^. The case presented highlights the complex intersection of congenital aortic stenosis and the long-term implications of FH on both aortic and coronary pathology, as demonstrated by the patient’s initial need for a Ross procedure and later coronary revascularization.

In one small retrospective study reporting outcomes after CABG in 101 patients with FH, single or multiple arterial grafting was superior to only vein grafting. However, there was no survival benefit to multiple arterial grafting over a single artery^6^.

The best candidates for the Ross procedure are younger adults with isolated aortic stenosis, preserved aortic root architecture, no significant aortic root dilation, and no evidence of diffuse aortic or coronary atherosclerosis at the time of surgery. FH patients with advanced aortic root calcification, supravalvular aortic involvement, or diffuse coronary disease pose a higher risk of early autograft dysfunction and may be better served with alternative valve replacement options^14^. Mechanical prostheses remain durable but require ongoing anticoagulation, whereas bioprosthetic valves deteriorate over a period of 10–15 years. Transcatheter aortic valve replacement (TAVR) has also been described in FH with promising efficacy, particularly for older individuals or those at high surgical risk, but data remain limited for younger cohorts. Comprehensive imaging of the aortic root, assessment of coronary anatomy, and evaluation for concomitant hyperlipidemia-induced tissue degeneration should guide surgical planning^15^.

The mean time to reoperation after Ross procedure is approximately 12.6 years from the index procedure, with survival after reoperation approximately 80% at 20 years^7^. The clinical team must weigh the benefits of the Ross procedure against the potential need for future reoperations, and alternative surgical approaches may need to be explored. Comprehensive management, including early and aggressive lipid-lowering therapy, remains critical in mitigating the cardiovascular risks associated with FH.

FH patients undergoing the Ross procedure, therefore, represent a unique population where the long-term durability of the autograft may be compromised due to the pathologic processes driving both valvular and vascular disease. This case illustrates the need for careful consideration of the Ross procedure in FH patients, as they may face higher rates of reoperation compared to those without FH. Even with aggressive lipid-lowering therapies, patients with FH often experience progressive CAD at a young age^8^. However, lifelong adherence to lipid-lowering therapy reduces the risk of coronary events and slows the progression of CAD^9^. Studies have shown an average 21% reduction of major cardiovascular events for every one mmol/L reduction in LDL-C^10^.

In this case, the patient required stenting of the LAD and LCx arteries, followed by a three-vessel CABG. This progression underscores the relentless nature of CAD in FH despite modern therapeutic interventions. Early SVG failure in the setting of FH is predominantly attributed to the lifelong exposure to markedly elevated LDL cholesterol. Excess LDL-C accumulates within the vein graft wall after implantation, leading to accelerated development of intimal thickening, endothelial dysfunction, and lipid-rich atherosclerotic plaque^11,12^. These pathological changes trigger graft narrowing and, ultimately, occlusion, often within the first year after surgery despite aggressive lipid-lowering therapy. The persistent atherogenic environment of FH creates a strong stimulus for early and diffuse SVG disease.

Careful preoperative planning is needed in patients who require redo cardiac surgery after the Ross procedure. As in any root replacement, care is needed in dissection for cross-clamp and proximal anastomoses placement due to adhesions in the area around and between the aorta and pulmonary artery and near the coronary buttons. It should be noted that peripheral cannulation was undertaken to reduce the risk of injury during re-sternotomy in the case presented. However, innovative strategies are needed to improve safety in reoperations^13^, especially in this patient population, which will require higher rates of reintervention.

### What have we learned?

• Patients with familial hypercholesterolemia, coronary artery disease, and complex aortic disease require lifelong multidisciplinary management with cardiac surgery and interventional cardiology.
• There is a high risk of treatment failure with revascularization in this patient population due to the rapid progression of CAD, and strict medication adherence is paramount.
• There should be careful consideration of the need for subsequent cardiac surgery in young patients with familial hypercholesterolemia undergoing the Ross procedure. Optimal coronary revascularization strategies in these complex patients are challenging and require further research.

## Informed consent

As per JH Medicine Institutional Review Board, informed consent is not required for a case report. There are no images that are particularly unique and would be identifying for the report.

## Conflict of interest

The authors do not have any conflict of interest.

## Funding disclosures

The authors have nothing to disclose.

## Glossary of abbreviations

CABG: Coronary Artery Bypass Grafting; CTA: Computed Tomography Angiography; HTN: Hypertension; CAD: coronary artery disease; NSTEMI: Non ST elevation Myocardial Infection; PCI: Percutaneous Catheter Intervention; LAD: Left Anterior Descending; LCx: Left Circumflex; BMI: Body Mass Index; D1: First Diagonal Artery; D2: Second Diagonal Artery; OM1: First Obtuse Marginal Artery; OM2: Second Obtuse Marginal Artery; LC: Left Coronary; LVEDP: Left ventricular end-diastolic pressure; CPB: cardiopulmonary bypass; LDL-C: Low densitiy lipoprotein cholesterol; RA: Right Atrium; LA: Left Atrium; RV: Right Ventricle; LV: Left Ventricle; LVEF: Left Ventricular Ejection Fraction; SVG: Saphenous Venous Graft; POD: Post Operative Day; RCA: Right Coronary Artery.
